# Hyaluronic acid based extracellular matrix regulates surface expression of GluN2B containing NMDA receptors

**DOI:** 10.1038/s41598-017-07003-3

**Published:** 2017-09-08

**Authors:** Barbara Schweitzer, Jeet Singh, Anna Fejtova, Laurent Groc, Martin Heine, Renato Frischknecht

**Affiliations:** 1 Leibniz Institute for Neurobiology, Dept. for Neurochemistry and Molecular Biology, Brenneckestr. 6, Magdeburg, 39118 Germany; 2 RG Presynaptic Plasticity, Leibniz Institute for Neurobiology, Magdeburg, Germany; 3 Department of Psychiatry and Psychotherapy, University Hospital, Friedrich-Alexander-University Erlangen-Nuremberg, Erlangen, Germany; 4 Interdisciplinary Institute for Neuroscience, UMR 5297, 33000 Bordeaux, France; 5 RG Molecular Physiology, Leibniz Institute for Neurobiology, Brenneckestr. 6, 39118 Magdeburg, Germany; 6 Center for Behavioral Brain Sciences, D-39118, Magdeburg, Germany; 7 Department of Biology Animal Physiology, University of Erlangen-Nuremberg, Staudtstrasse 5, 91058 Erlangen, Germany

## Abstract

Cortical areas of the juvenile rodent brain display a high degree of structural and functional plasticity, which disappears later in development. Coincident with the decline of plasticity 1) the hyaluronic acid-based extracellular matrix (ECM) of the brain, which stabilizes synapses and neuronal circuit is formed and 2) N-methyl-D-aspartate subtype of ionotropic glutamate receptors (NMDARs) implied in synaptic plasticity switch from mainly GluN2B to GluN2A subunit-containing receptors. Here we tested the hypothesis that ECM influences the NMDAR subunit composition in dissociated neuronal cultures. Experimental removal of ECM using hyaluronidase induced an increase in surface expression of GluN2B. This was due to decreased endocytosis of surface GluNB-containing receptors. We further found a reduction in phosphorylation at Tyr1472, which negatively regulates their binding to the endocytotic AP2 complex. We propose that maturation of ECM could induce switch in NMDAR composition necessary for normal adult synaptic plasticity and that increased expression of GluN2B contributes to rejuvenation of plasticity after ECM removal *in vivo*.

## Introduction

During early postnatal development cortical areas of rodents exhibit a high degree of structural and functional plasticity, which declines during adolescence as the adult form of the extracellular matrix (ECM) is formed. The ECM of the brain surrounds cell-bodies, apical dendrites and enwraps synaptic contacts^1^. It consists of chondroitin sulfate proteoglycans (CSPGs) that form an extracellular proteineous meshwork around hyaluronic acid, the ECMs backbone^2^. The ECM affects synaptic plasticity on multiple levels. ECM removal impaired long-term potentiation (LTP), long-term depression (LTD)^3, 4^ but improved LTP in the visual cortex *in vivo* ^5^. Further, ECM removal altered short-term plasticity (STP) and affected surface diffusion of AMPA receptors^6^. In addition, CSPGs restrict structural as well as regenerative plasticity in part by inhibiting ß1-containing integrins^7–9^. Accordingly enzymatic removal of the ECM improved regenerative and structural plasticity, induced higher cognitive flexibility and restored so-called critical period in the visual cortex in the adult and thus a form of experience dependent plasticity present in the developing animal^1, 10^.

N-methyl-D-aspartate receptors (NMDARs) are a subtype of ionotropic glutamate receptors that are crucial for many forms of synaptic plasticity, learning and memory formation and synaptic development which emerges from their Ca^2+^ permeability (for a review see. ref. 11). They are assembled as tetramers composed of two GluN1 subunits and two GluN2 or GluN3 subunits. Recently it has been found that NMDARs assemble as tri-heteromers that differ in composition depending on developmental stage^12^. In the hippocampus and cortex, GluN2A and GluN2B are the predominant subunits indicating their importance for synaptic function and plasticity^13–15^. Depending on their respective abundance at the synapse they differentially influence synaptic plasticity, which results from their diverse kinetic properties and binding partners to their cytoplasmic tails^12^. During development there are predominantly GluN2B containing NMDA receptors (GluN2B-NMDARs) at the synapse^16, 17^. Interestingly, concurrent with the closing of the critical period, GluN2A expression rises, which results in an increase in the synaptic GluN2A/GluN2B ratio^18–21^. The juvenile composition of NMDAR can be restored in rats by visual deprivation and subsequently ocular dominance plasticity is reinstalled^22^. Thus GluN2B and ECM are converse regulated key players during critical period plasticity. Here we investigated the impact of the ECM on GluN2B surface expression and trafficking. We found 12 hours after enzymatic ECM removalwith hyaluronidase (Hya) larger GluN2B mediated synaptic currents, which is due to increased surface expression of GluN2B. This effect was β1-integrin dependent and is accompanied by phosphorylation of the YEKL motif on the C-terminus of the GluN2B subunit, which has been shown to prevent receptor endocytosis. Thus, we suggest that ECM removal shifts synaptic GluN2A/GluN2B ratio towards their juvenile composition, which may in part account for the restoration of developmental plasticity in the adult after ECM removal.

## Methods

### Ethics statement

All experimental procedures were carried out in accordance with the EU Council Directive 86/609/EEC and were approved and authorized by the local Committee for Ethics and Animal Research (Landesverwaltungsamt Halle, Germany).

### Primary neuronal cultures

Primary neuronal cultures were prepared from E18 Wistar rats. Hippocampal cultures were made as described previously^23^. Briefly, cells were plated at a density of 3 × 10^5^ cells per ml on poly-L-lysine pre-coated 18 mm cover slips. Cultures of cortical neurons were plated at a density of 1 × 10^5^ cells per ml on poly-D-lysine pre-coated cover slips in 24 well dishes. For quantitative Western blot the cells were plated at a density of 5 × 10^5^ cells per ml in a 6well dish. The Cultures were maintained in serum-free neurobasal medium (Invitrogen) and kept at 37 °C in 5% CO_2_ for 11–28 DIV (days *in vitro*).

### Antibodies and drugs

The following commercial antibodies were used for Immunocytochemistry (ICC) and Western blot (WB) in the concentrations indicated: rabbit (rb) antibodies against GluN2B (alomone labs; ICC live staining: 1:200, fixed staining.: 1:1000; endocytosis assay: 1:25), antibodies against GluN2A (alomone labs; 1:500) and GluN1 (Synaptic Systems; 1:200), anti pGluN2BTyr1472 antibody (AAT Bioquest; WB 1:500); mouse (ms) antibodies against GluN2B (NeuroMab; WB 1:500), PSD-95 (NeuroMab; ICC 1:1000), Map2 (Sigma-Aldrich; ICC 1:2000), β3-tubulin (Synaptic System; WB 1:1000); rat antibody against β1-integrin CD29 (BD Pharmingen; 1:25).

Fluorescently labelled secondary antibodies that were used for ICC against rabbit, mouse, guinea pig were purchased from Invitrogen conjugated with either Alexa 488, 568, 647 (1:1000) or from Dianova conjugated with Cy3, Cy5 (1:1000). Fluorescently labelled secondary antibodies against ms, rb and guinea pig for quantitative immunoblotting were purchased from Invitrogen (ms Alexa Fluor 680, 1:20,000) and from Rockland (rb IRDye 800 W, 1:20,000).

Hyaluronidase (Hya, Sigma-Aldrich) was used at 100 units/ml, and TTX (0, 5 µM), CNQX (5 µM), Biccuculine (BCC) (10 µM), AP5 (10 µM), Ifenprodil (3 µM) was purchased from Tocris. All other chemicals and drugs were purchased from Sigma-Aldrich (USA).

### Electrophysiology

Patch clamp recordings were made at 33–35 °C using borosilicate pipettes to produce patch electrodes with resistances of 3–5 MΩ. Extracellular medium contained 145 mM NaCl, 5 mM KCl, 1 mM CaCl_2_, 10 mM HEPES and 10 mM D-glucose, 15 µM Glycine (pH 7.4). and 10 µM BCC, 5 µM CNQX. For the initial isolation of synaptic NMDAR mediated events we either added 2 mM CaCl_2_ and 0.5 mM MgCl_2_ and antagonists as 10 µM APV and 3 µM Ifenprodil, as will be indicated in the results. For the spontaneous network activity driven excitatory synaptic currents (sEPSCs) a standard pipette solution was used, which contained 140 mM potassium gluconate, 2 mM MgCl_2_, 4 mM NaATP, 0.1 mM EGTA, 10 mM HEPES, 10 mM phosphocreatine, 0.4 mM GTP and 10 µM QX314 (pH 7.25). Global block of sodium channels with 0.5 µM TTX in the extracellular solution did drastically reduce the amplitude of NMDAR evoked mEPSCs, which were difficult to analyse, particular in respect to kinetic properties of the NMDAR and rejected for further analysis (data not shown). Probing the effect of blocking network activity by 0.5 µM TTX on AMPAR mediated EPSCs did result in a loss of burst like activity, but did not alter dramatic the amplitude or kinetic of AMPAR mediated EPSCs outside burst activity (Supplementary Figure S1A,B). Recordings in voltage clamp mode were performed at holding potential of −70 mV with an EPC10 double patch-clamp amplifier (HEKA Electronics). Data were acquired and stored using Patch Master Version 2.11 (HEKA Electronics, Lambrecht, Germany). Peak detection was done with Minianalysis (Synaptosoft) and analysed with GraphPad Prism software.

### Immunocytochemistry

Living neurons were incubated with GluN2B antibody in culture medium at 37 °C or at 4 °C for 20 min, fixed in 4% paraformaldehyde (w/v) in PBS for 5 min and subsequently incubated for 30 min incubation in blocking solution (10% FCS in PBS, 0,1% Glycin, 0,1% Triton X-100). Further primary antibodies were incubated for 90 min at RT. After three washing steps with PBS cells were incubated with secondary antibody for 1 h at RT. Cells were washed and mounted in Mowiol. Preparations were kept at 4 °C until examination. Images were acquired on a Zeiss axioplan fluorescence microscope and processed for quantitative analyses with ImageJ (US National Institutes of Health). Quantification of synaptic GluN2B intensity was done by OpenView Software by Noam Ziv.

Immunostainings of the total amount of GluN2B followed basically the same protocol but the GluN2B antibody was applied after fixation and permeabilization.

### Endocytosis assay

Living neurons were incubated with GluN2B antibody in culture medium at 4 °C for 30 min. Cells were washed and placed back into the culture dish at 37 °C for 30 min. Subsequently cells were incubated with secondary antibody at 4 °C for 10 min; washed and fixed with 4% paraformaldehyde (w/v) in PBS for 5 min followed by a 1 h incubation in blocking solution. Cells were incubated for 90 min at RT with primary antibody against Map2. After three washing steps with PBS cells were incubated with secondary antibody against ms 647 and rb 488 to stain the dendrites and the endocytosed GluN2B receptors respectively. Cells were washed and mounted in Mowiol.

### Acute hippocampal slices

Rat brain was rapidly removed and immersed in oxygenated ice-cold ACSF (125 mM NaCl, 2,5 mM KCl, 1,25 mM NaH_2_PO_4_, 25 mM NaHCO_3_, 2 mM CaCl_2_, 1 mM MgCl_2_, 25 mM glucose). Hippocami were isolated and transverse hippocampal slices (350 µm) were prepared using a Vibratome (The Vibratome Company). The slices recovered in aCSF at 32 °C for 1 h 45 min. All the solutions were oxygenated by continuous bubbling with 95% O_2_/5% CO_2_. Slices from both hippocampi were pooled and randomly separated for the different treatment groups (3 slices per group). Treatment duration: 3 h. After treatment the slices were transferred to Lysis Buffer (10 mM Tris-HCl, pH 7.4, 150 mM NaCl, 2% SDS, 1% deoxycholate, 1% Triton X-100, supplemented with Complete Protease Inhibitor Cocktail (Roche), PhosSTOP Phosphatase Inhibitor Cocktail (Roche) and 1% NaVO_3_), triturated and stored at −80 °C until use for SDS-Page and Western-Blot.

### SDS-Page, Western Blotting (WB)

Cell lysates of acute hippocampal slices or cortical cultures were separated using one-dimensional SDS-PAGE and then electrotransferred to Millipore Immobilon-FL PVDF membranes. Western blots were blocked with 5% BSA in TBS-T and subsequently incubated with primary antibodies (diluted in TBS containing 0.1% Tween 20 and 2% BSA) and afterwards with fluorescently labeled secondary antibodies (diluted in TBS containing 0.1% Tween 20). For detection of phosphorylated GluN2B all solutions containing BSA were supplemented with PhosSTOP Phosphatase Inhibitor Cocktail (Roche) and 1% NaVO_3_. Immunodetection was performed using an Odyssey Infrared Scanner (LICOR). Quantification was done using ImageJ.

## Results

### Hya treatment enhances GluN2B-NMDAR mediated sEPSCs

In order to analyze the influence of the ECM on synaptic transmission we performed patch clamp recordings of control (Ctl) and Hya treated dissociated hippocampal cultures at DIV 21–24. At this time-point the ECM is fully developed in culture^6, 24–26^. In previous experiments we could not find any differences in mEPSCs amplitude and frequency between control and Hya treatment^6, 27^. In the presence QX314 (in the pipette solution) and MgCl_2_ (0.5 mM in the extracellular solution; holding membrane potential −70 mV) we did not find any changes in amplitude or kinetics of network activity driven spontaneous EPSCs (sEPSC) outside regular bursts of activity, suggesting AMPA receptor composition and abundance to be unaffected as we reported previously^6, 27^ (Supplementary Figure S1A). Blocking sodium channel activity not only by perfusion of the patched cell with QX314 (10 µM) via the patch pipette, but applying 0.5 µM TTX in the extracellular solution did remove burst like activity. Comparing the sEPSCs outside burst - like activity (QX314 in the patch pipette) and mEPSCs (QX314 in the patch pipette and TTX in the bath solution) did not show major differences in their amplitudes and kinetics.

**Figure 1:**
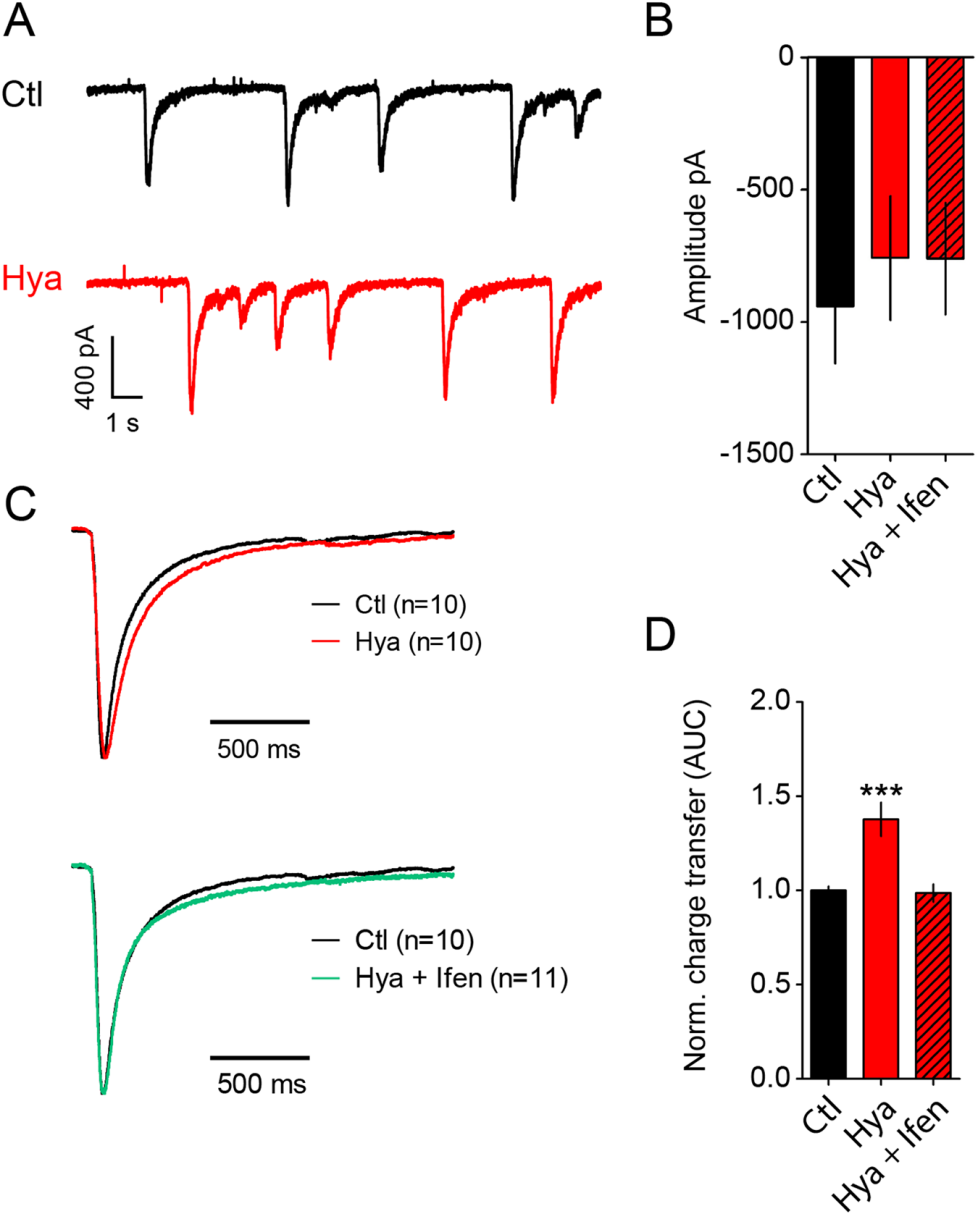
ECM removal enhances GluN2B-NMDAR mediated synaptic currents. (**A**) Example traces of NMDAR - mediated sEPCSs before and after Hya treatment in dissociated hippocampal cultures DIV21-24. (**B**) Amplitudes of single peaks show no significant differences between Hya treated or Hya plus Ifenprodil treated cultures (Ctl, −905.5 ± 179.4, n = 10; Hya, −776.2 ± 174.8, n = 10; Hya + Ifen, −758.2 ± 161.7, n = 11; average ± SEM; One-way ANOVA, P = 0.7991). (**C**) Average of single peaks before and after Hya treatment and after Ifenprodil application. Normalization of the amplitude illustrates the increased decay-time after Hya treatment (red line) in comparison to Ctl (black line). This can be restored after Ifenprodil application (green line). Ctl traces are identical. (**D**) Quantification of the area under the curve (AUC) of averaged and normalized events (left), which represent the total charge transfer revealed bigger charge transfer after ECM removal, which was reduced to control levels after blocking GluN2B-NMDAR with Ifen (Ctl, 1 ± 0.02, n = 10; Hya, 1.38 ± 0.09, n = 10; Hya + Ifenprodil, 0.98 ± 0.05, n = 11; average ± SEM; One-way ANOVA, P < 0.0001, Dunnett’s Multiple Comparison Test, ***P < 0.05).

In order to measure the contribution of NMDAR-mediated currents we measured sEPSCs in the absence of TTX and extracellular Mg^2+^ and adding 15 µM Glycin to the extracellular solution. The contribution of AMPARs was blocked by CNQX (5 µM) in the bath solution. Under this condition we observed events that showed highly variable amplitudes of 0.4–1.6 nA and lasted for about 1.5 s (Fig. 1A). These events were entirely driven by NMDAR activation and disappeared completely after application of APV (Supplementary Figure S1C). Adding TTX in the extracellular solution to prevent network activity did block these currents. The identification of NMDAR - mediated mEPSCs under this condition was strongly biased by the massive reduction in the frequency of clearly identifiable events provoked by NMDAR activation (data not shown). In order to identify a possible contribution of NMDAR before and after matrix digestion we explored the kinetic properties of NMDAR driven sEPSCs, which had a monotonic rise time of the current. Thus, comparing the time course of the current decay for events normalized to their maximal amplitude under the different conditions should indicate a contribution of different NMDAR populations, since in particular their desensitization properties differ substantially between GluN2A and GluN2B containing receptors^12^. NMDAR-mediated sEPSCs did not show any changes in amplitudes after Hya treatment, which remained as variable as under control conditions (Fig. 1A,B) and is most likely biased by network interactions. However, the relative charge transfer of the single events showed an increase of 38% in Hya-treated cells in comparison to untreated control cells (Fig. 1D). Comparison of normalized amplitudes between the two treatments showed that the increase in charge transfer was due to longer decay times of the currents (Fig. 1C). Treatment with Ifenprodil (Ifen, 3 µM) reduced the charge transfer to control level, indicating that this effect was specific to GluN2B-containing NMDAR (Fig. 1C,D), prolonging the decay time of the evoked currents. Due to the difficulties to isolate single synaptic vesicular events in our electrophysiological recordings we used immunhistochemical and biochemical approaches to investigate the surface population of NMDAR in more detail.

### Hya treatment does not affect total protein amount of GluN2B

**Figure 2:**
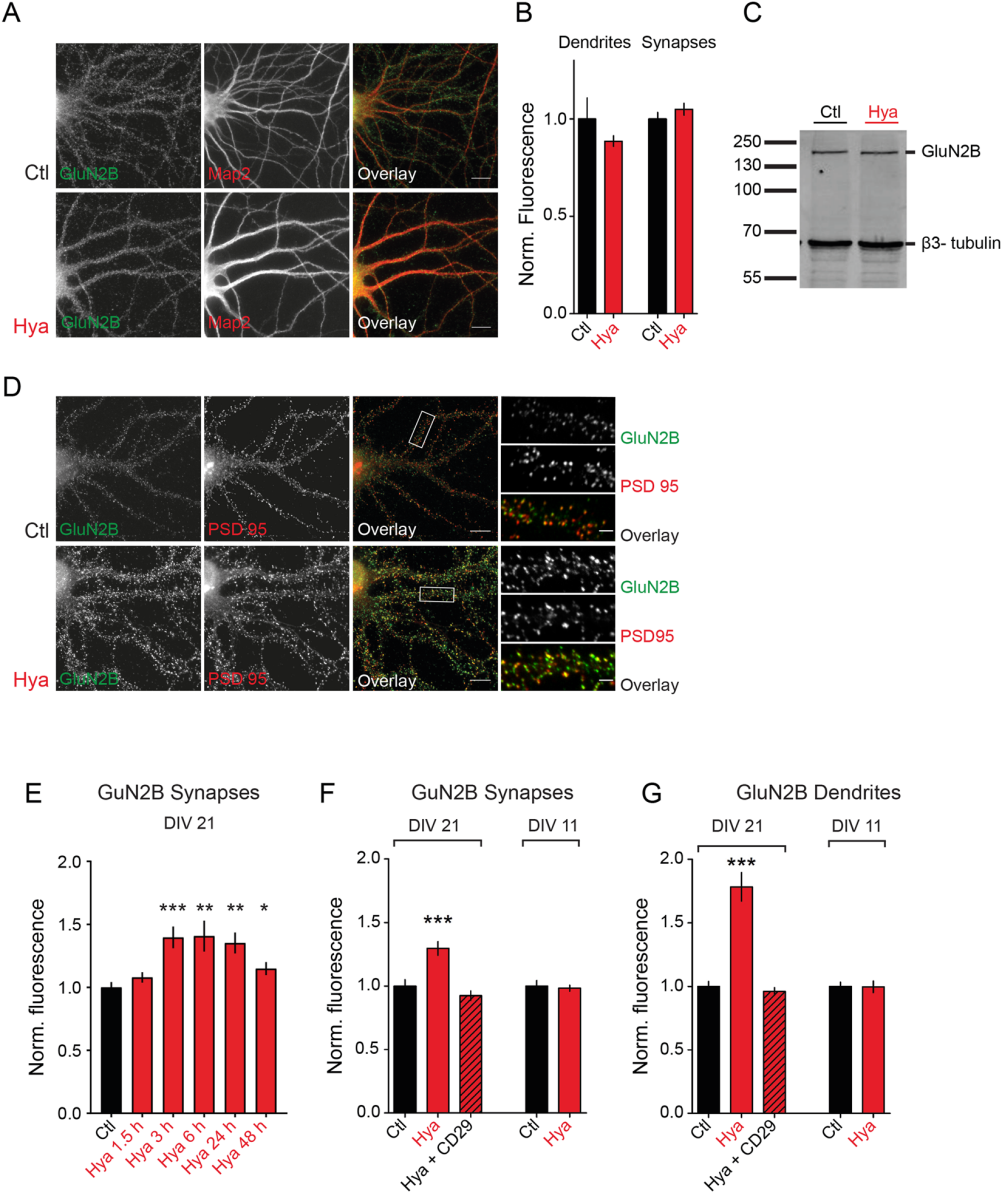
ECM removal leads to increased surface expression of GluN2B in a β1 - integrin dependent manner. (**A**) Dissociated hippocampal cultures were treated with Hya over night and stained against the total amount of GluN2B and the dendritic marker Map2 (scale bar: 10 μm. (**B**) Total GluN2B expression is not affected by ECM removal (Dendrites: Ctl 1 ± 0.10, n = 30; Hya 0.89 ± 0.03, n = 30, P = 0.31; Synapses: Ctl: 1 ± 0.03, n = 30; Hya: 1.05 ± 0.03, n = 30, P = 0.27; average ± SEM; unpaired t-test). (**C**) Quantitative WB of lysed cortical cultures (DIV21) pretreated with Hya over night show no significant change in GluN2B immunoreactivity. (**D**) Dissociated hippocampal cultures at DIV21-24 were treated with Hya over night and stained against surface GluN2B (green) and the synaptic marker PSD-95 (scale bar: 10 μm). (**E**) Synaptic GluN2B surface expression at various time points after Hya treatment (Ctl: 1 ± 0.04, n = 24; Hya 1,5 h: 1.08 ± 0.04, n = 22, P = 0.76; Hya 3 h: 1.40 ± 0.09, n = 30, P = 0.0001; Hya 6 h: 1.41 ± 0.13, n = 9, P = 0.002; Hya 12 h: 1.35 ± 0.08, n = 8, P = 0.01; Hya 48 h: 1.18 ± 0.05, n = 8, P = 0.04 average ± SEM; One way-ANOVA, Dunnett’s Multiple Comparison Test). (**F,G**) GluN2B surface expression at synapses and dendrites increases after ECM degradation and can be restored by simultaneous application of the β1-integrin function blocking antibody CD29. (**F**) Synapses: Ctl: 1.0 ± 0.05, n = 68; Hya: 1.3 ± 0.05, n = 70; Hya + CD29: 0.93 ± 0.03, n = 51. (**G**) Dendrites: Ctl 1.00 ± 0.04, n = 36; Hya 1.78 ± 0.11, n = 35; Hya + CD29 0.96 ± 0.03, n = 34; average ± SEM; One-way ANOVA, P < 0.0001, Dunnett’s Multiple Comparison Test, ***P < 0.001). No ECM dependent regulation in hippocampal cultures at DIV11 (Synpases: Ctl: 1.00 ± 0.04, n = 25, Hya: 0.98 ± 0.03, n = 24, average ± SEM, unpaired t-test, P = 0.7341; Dendrites: Ctl: 1,000 ± 0.03, n = 39, Hya: 0.99 ± 0.05, n = 40; average ± SEM, unpaired t-test, P = 0.9488).

Next we wondered whether increased GluN2B-NMDAR mediated synaptic currents are due to an overall elevated GluN2B expression level. Therefore, we performed immunocytochemistry on dissociated cortical neurons under permeabilizing conditions to label surface and intracellular GluN2B-NMDARs (Fig. 2A). No differences were detectable between Hya and control group, neither at synapses nor in the whole dendritic compartment (Fig. 2A,B). Further, we performed WB analysis of dissociated cortical neurons and compared Hya treated to control cells (Fig. 2C). However, we found no difference in the total expression level of GluN2B. These results suggest that ECM removal can increase synaptic transmission via GluN2B-NMDAR without affecting the total amount of GluN2B.

### Increased surface expression of GluN2B after ECM removal depends on β1-integrin

Next we investigated whether surface expression of GluN2B-NMDARs was altered. For this purpose we performed immunocytochemistry experiments on living, non-permeabilized hippocampal neurons at several time points after Hya treatment (Fig. 2D,E). Surface GluN2B subunits were labeled using an antibody against their extracellular N-terminus. Synaptic receptors were defined by co-labeling with the synaptic protein PSD-95 (Fig. 2D). Intensity analysis revealed an increase of over 25% of synaptic GluN2B 3 h after Hya treatment (Fig. 2E), which remained elevated for 48 h, although to a lesser extent. In addition we found a slight increase of surface expression of the GluN1 subunit but not GluN2A (Supplementary Figure S2A,B). Measuring surface expression of GluN2B on whole dendrites using Map2 staining as mask for quantification, revealed an increase of about 78% of surface GluN2B after ECM removal (Fig. 2G). This indicates a general increase in surface expression, which is not synapse specific but affects the extrasynaptic population of GluN2B as well. To test for unspecific effects of Hya on GluN2B-NMDAR surface expression we treated hippocampal neurons at DIV11, a developmental stage in which neuronal culture do not show a fully developed ECM^24^. As expected there was no significant difference of GluN2B-NMDAR surface expression between treated and untreated cells neither at synapses (Fig. 2F) nor at dendrites (Fig. 2G). This clearly indicates that increased GluN2B surface expression is a specific effect of the ECM degradation.

Candidates transducing extracellular ECM-derived signals into the cell are integrins. To test whether the ECM dependent increase of surface GluN2B-NMDAR depends on integrin signaling we applied a β1-integrin function-blocking antibody (CD29) during ECM removal. Interestingly this inhibited the up regulation of surface GluN2B at synapses as well as on dendrites (Fig. 2F and G). Altogether, these data suggest that the ECM removal increased GluN2B surface abundance by activation of a β1-integrin dependent signaling mechanism.

### Hya treatment decreases endocytosis of GluN2B

**Figure 3:**
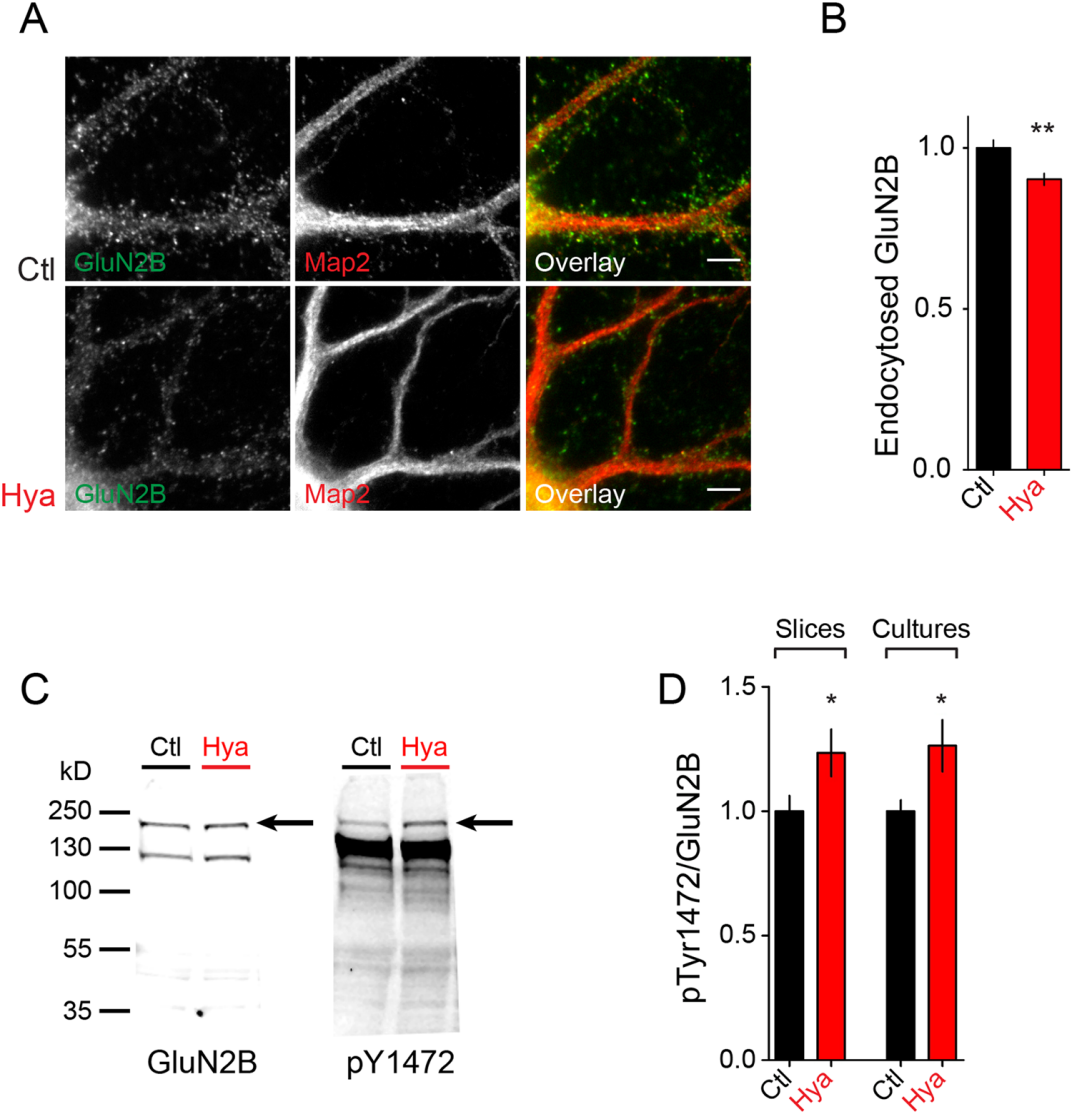
ECM digestion increases p1472-GluN2B level and decreases the endocytosis of GluN2B. (**A**)Dissociated hippocampal cultures at DIV21-24 were treated with Hya over night and endocytosed GluN2B (green) was quantified using Map2 staining as mask (red). (**B**) There is less endocytosis of GluN2B after ECM removal within 30 minutes (Ctl 1.00 ± 0.02, n = 79; Hya 0.9 ± 0.02, n = 80; average ± SEM, Unpaired t-test, **P = 0.0015. Scale bar: 5 µm). (**C**) Quantitative WB from lysates of acute hippocampal slices treated with Ctl or Hya probed with an antibody against pGluN2B pTyr1472 (AP2 binding site) and GluN2B. (**D**) Quantification of WB of acute hippocampal slices and cortical cultures (DIV 21–24) revealed that the amount of phosphorylated GluN2B, normalized to the total amount of GluN2B, is increased after Hya treatment (overnight for cultures, 3 h for slices; slices: Ctl 1.00 ± 0.06, n = 4; Hya 1.23 ± 0.09, n = 4; cultures: Ctl 1.00 ± 0.05, n = 9; Hya 1.26 ± 0.1, n = 9; Unpaired t-test, cultures: P = 0.0332, slices P = 0.0837, ***P < 0.0001).

The finding that the total amount of GluN2B expression was unaltered while surface expression was increased suggested altered GluN2B-NMDAR trafficking after Hya treatment. Therefore, we tested whether GluN2B-NMDAR endocytosis was altered after Hya treatment and performed an endocytosis assay in dissociated cortical cultures. We incubated cells with GluN2B antibodies at 4 °C to block membrane trafficking and subsequently placed cells into the incubator at 37 °C to allow for endocytosis for 30 min. We then differentially labeled the remaining surface exposed GluN2B and the endocytosed GluN2B antibodies and measured the fluorescence intensity of endocytosed receptors (Fig. 3A). Interestingly we found significantly less endocytosed GluN2B within 30 min after ECM removal (Fig. 3B). This points toward a longer surface residual of the receptor, which over time leads to a surface accumulation of GluN2B. To test whether regulation was specific for GluN2B we performed the endocytosis assay using GluN1 and GluN2B antibodies (Supplementary Figure S2C,D). We found slight decrease of GluN1 while GluN2A endocytosis of GluN2A remained unchanged after Hya treatment.

Surface abundance of GluN2B has been reported to be regulated via a specific phosphorylation of the clathrin adaptor protein adaptor protein 2 (AP2) binding site, the tyrosin residue Y1472. Phosphorylation of Y1472 prevents binding of AP2 and inhibits endocytosis of GluN2B^28^. To test for an involvement of this phosphorylation in the observed increased surface expression of GluN2B we performed semi- quantitative WB experiments from cortical cultures and acute hippocampal slices (Fig. 3C, D). We found that after ECM removal in both cortical cultures and acute hippocampal slices the amount of pY1472-GluN2B is increased (Fig. 3D). Thus, Hya treatment leads to gradual accumulation of surface GluN2B-NMDAR by promoting phosphorylation of Y1472 and delaying its endocytosis.

## Discussion

In this study we found that enzymatic ECM removal lead to a β1-integrin-dependent increase in GluN2B surface expression without changing its total expression level. This may be due to increased phosphorylation of Tyr1472 within the “YEKL”-motif of GluN2B, which prevents its binding to the AP-2 complex and thus its endocytosis, leading to a slow surface accumulation of GluN2B.

Dissociated hippocampal cultures form ECM within the first 2–3 weeks *in vitro* in activity-dependent manner with similar composition as found *in vivo* ^24, 26^. We have previously reported that in this culture system enzymatic ECM removal leads to altered AMPAR diffusion on excitatory but not inhibitory neurons without affecting AMPA or GluN2A surface expression or basal synaptic transmission^6, 27^. This is in line with our present findings, where we observed no difference in AMPA driven synaptic transmission after Hya treatment (Supplementary Figure S1A). However, when we isolated NMDAR currents, we found elevated GluN2B-NMDAR dependent charge transfer due to increased GluN2B surface expression. GluN2B-NMDAR and GluN2A-NMDAR differ in their trafficking and mobility. GluN2B containing NMDARs undergo more frequent endocytosis than GluN2A containing NMDARs in mature neuronal cultures^29^. This difference is owed to the cytoplasmic domains of GluN2A and GluN2B, which are least conserved regions among the NMDAR subunits^12^. Regulation of GluN2B surface levels is attributed to phosphorylation of specific regions unique to GluN2B subunit. CKII dependent phosphorylation of S1480 within the MAGUK/PDZ binding motif leads to a disruption of the interaction of MAGUKs with GluN2B and to lateral movement of GluN2B to extrasynaptic sites where the endocytotic zones are located. This disruption triggers the dephosphorylation of Y1472 and subsequently to the internalization of GluN2B by binding of AP-2^30^. It is well established that phosphorylation of Y1472 within the “YEKL”-motif of GluN2B regulates surface expression and endocytosis of GluN2B by impairing binding to the AP-2 complex and thus prevents endocytosis^28, 31–35^. In line with this we found increased phosphorylation of Y1472 within the “YEKL”-motif after ECM removal and a decreased rate of endocytosis (Fig. 3A,B). As responsible kinases the Src kinase family member Fyn and Src have been suggested^32, 36^. Interestingly, it has been shown that application of integrin ligand peptides leads to a rapid increase in tyrosine phosphorylation by src family kinases. This was followed by elevated NMDAR mediated synaptic responses^37, 38^. Our findings that Integrin blocking antibodies prevented surface accumulation of GluN2B (Fig. 2F, G) fits to this scenario and further supports the idea of an integrin dependent regulation of NMDAR trafficking.

Integrins are present at the cell surface in different states: an inactivated state, an activated state and a ligand bound state^39, 40^. Several studies showed that CSPGs, eg. Aggrecan and Versican interact with β1-integrins and thereby decrease or increase phosphorylation state of Y397-FAK, respectively^41, 42^. It was suggested that CSPGs keep integrins in the inactive conformation and thus prevent integrin signaling^41^. In line with this idea chondroitinase ABC (chABC) treatment, which has a similar effect on ECM as Hya, leads to increased integrin signaling associated with neurite outgrowth in sensory neurons^41^. CSPG removal by chABC also enhances β1-integrin activation and pY397- FAK level in the hippocampus^7^. Autophosphorylation of Y397-FAK leads to association with Src, resulting in an activation of both kinases^43, 44^. In our study we could show that Hya increased surface expression of GluN2B. Important to note is that the Hya used in this study has an intrinsic chondrotinase activity and thus Hya and chABC treatment abolish CS and thereby remove inhibition of integrin. However, the specific ligand leading to β1-integrin signaling remains to be determined. Candidates like reelin have been suggested to play an important role in regulating NMDAR trafficking^45^.

Interestingly, the appearance of the HA-based ECM marks the end of the critical periods in the visual cortex during which topographic maps are formed. It is long time known that removal of the ECM by enzymatic treatment reinstalls critical periods and in addition loosened fear learning and enhanced cognitive flexibility^1, 10, 46^. Interestingly, GluN2B is down regulated during critical periods and dark rearing of animals prolongs critical periods and prevents GluN2B regulation as well as ECM formation^1, 47, 48^. Although, there is a clear correlation between ECM appearance and GluN2B regulation, there was to date no evidence for a functional between the two processes connection. Here we provide evidence that ECM removal influences GluN2B surface expression in a β1-integrin dependent manner, which may be in part responsible for the reopening of juvenile forms of plasticity after ECM removal.

## Electronic supplementary material

Supplementary information

## References

[1] Pizzorusso T (2002). Reactivation of ocular dominance plasticity in the adult visual cortex. Science.

[2] Frischknecht R, Seidenbecher CI (2008). The crosstalk of hyaluronan-based extracellular matrix and synapses. Neuron Glia Biol.

[3] Bukalo O, Schachner M, Dityatev A (2001). Modification of extracellular matrix by enzymatic removal of chondroitin sulfate and by lack of tenascin-R differentially affects several forms of synaptic plasticity in the hippocampus. Neuroscience.

[4] Kochlamazashvili G (2010). The extracellular matrix molecule hyaluronic acid regulates hippocampal synaptic plasticity by modulating postsynaptic L-type Ca(2+) channels. Neuron.

[5] de Vivo L (2013). Extracellular matrix inhibits structural and functional plasticity of dendritic spines in the adult visual cortex. Nat Commun.

[6] Frischknecht R (2009). Brain extracellular matrix affects AMPA receptor lateral mobility and short-term synaptic plasticity. Nat Neurosci.

[7] Orlando, C., Ster, J., Gerber, U., Fawcett, J. W. & Raineteau, O. Perisynaptic chondroitin sulfate proteoglycans restrict structural plasticity in an integrin-dependent manner. *J Neurosci* **32**, 18009–18017, 18017a, doi:10.1523/JNEUROSCI.2406-12.2012 (2012). 10.1523/JNEUROSCI.2406-12.2012 PMC6621736 23238717

[8] Pizzorusso T (2006). Structural and functional recovery from early monocular deprivation in adult rats. Proc Natl Acad Sci USA.

[9] Moon LD, Asher RA, Rhodes KE, Fawcett JW (2001). Regeneration of CNS axons back to their target following treatment of adult rat brain with chondroitinase ABC. Nat Neurosci.

[10] Happel MF (2014). Enhanced cognitive flexibility in reversal learning induced by removal of the extracellular matrix in auditory cortex. Proc Natl Acad Sci USA.

[11] Shipton OA, Paulsen O (2014). GluN2A and GluN2B subunit-containing NMDA receptors in hippocampal plasticity. Philos Trans R Soc Lond B Biol Sci.

[12] Paoletti P, Bellone C, Zhou Q (2013). NMDA receptor subunit diversity: impact on receptor properties, synaptic plasticity and disease. Nat Rev Neurosci.

[13] Ikeda K (1992). Cloning and expression of the epsilon 4 subunit of the NMDA receptor channel. FEBS Lett.

[14] Akazawa C, Shigemoto R, Bessho Y, Nakanishi S, Mizuno N (1994). Differential expression of five N-methyl-D-aspartate receptor subunit mRNAs in the cerebellum of developing and adult rats. J Comp Neurol.

[15] Monyer, H., Burnashev, N., Laurie, D. J., Sakmann, B. & Seeburg, P. H. Developmental and regional expression in the rat brain and functional properties of four NMDA receptors. *Neuron* **12**, 529–540, doi:0896-6273(94)90210-0 [pii] (1994). 10.1016/0896-6273(94)90210-0 7512349

[16] Kleinschmidt A, Bear MF, Singer W (1987). Blockade of “NMDA” receptors disrupts experience-dependent plasticity of kitten striate cortex. Science.

[17] Fox K, Sato H, Daw N (1989). The location and function of NMDA receptors in cat and kitten visual cortex. J Neurosci.

[18] Flint AC, Maisch US, Weishaupt JH, Kriegstein AR, Monyer H (1997). NR2A subunit expression shortens NMDA receptor synaptic currents in developing neocortex. J Neurosci.

[19] Sheng M, Cummings J, Roldan LA, Jan YN, Jan LY (1994). Changing subunit composition of heteromeric NMDA receptors during development of rat cortex. Nature.

[20] Hoffmann H, Gremme T, Hatt H, Gottmann K (2000). Synaptic activity-dependent developmental regulation of NMDA receptor subunit expression in cultured neocortical neurons. J Neurochem.

[21] Erisir A, Harris JL (2003). Decline of the critical period of visual plasticity is concurrent with the reduction of NR2B subunit of the synaptic NMDA receptor in layer 4. J Neurosci.

[22] He HY, Hodos W, Quinlan EM (2006). Visual deprivation reactivates rapid ocular dominance plasticity in adult visual cortex. J Neurosci.

[23] Kaech S, Banker G (2006). Culturing hippocampal neurons. Nat Protoc.

[24] John N (2006). Brevican-containing perineuronal nets of extracellular matrix in dissociated hippocampal primary cultures. Mol Cell Neurosci.

[25] Bikbaev A, Frischknecht R, Heine M (2015). Brain extracellular matrix retains connectivity in neuronal networks. Sci Rep.

[26] Dityatev A (2007). Activity-dependent formation and functions of chondroitin sulfate-rich extracellular matrix of perineuronal nets. Dev Neurobiol.

[27] Klueva J, Gundelfinger ED, Frischknecht RR, Heine M (2014). Intracellular Ca(2)(+) and not the extracellular matrix determines surface dynamics of AMPA-type glutamate receptors on aspiny neurons. Philos Trans R Soc Lond B Biol Sci.

[28] Roche KW (2001). Molecular determinants of NMDA receptor internalization. Nat Neurosci.

[29] Lavezzari G, McCallum J, Dewey CM, Roche KW (2004). Subunit-specific regulation of NMDA receptor endocytosis. J Neurosci.

[30] Chen BS (2012). SAP102 mediates synaptic clearance of NMDA receptors. Cell reports.

[31] Sanz-Clemente A, Matta JA, Isaac JT, Roche KW (2010). Casein kinase 2 regulates the NR2 subunit composition of synaptic NMDA receptors. Neuron.

[32] Nakazawa T (2001). Characterization of Fyn-mediated tyrosine phosphorylation sites on GluR epsilon 2 (NR2B) subunit of the N-methyl-D-aspartate receptor. J Biol Chem.

[33] Lavezzari G, McCallum J, Lee R, Roche KW (2003). Differential binding of the AP-2 adaptor complex and PSD-95 to the C-terminus of the NMDA receptor subunit NR2B regulates surface expression. Neuropharmacology.

[34] Prybylowski K (2005). The synaptic localization of NR2B-containing NMDA receptors is controlled by interactions with PDZ proteins and AP-2. Neuron.

[35] Cheung HH, Gurd JW (2001). Tyrosine phosphorylation of the N-methyl-D-aspartate receptor by exogenous and postsynaptic density-associated Src-family kinases. J Neurochem.

[36] Zhang S, Edelmann L, Liu J, Crandall JE, Morabito MA (2008). Cdk5 regulates the phosphorylation of tyrosine 1472 NR2B and the surface expression of NMDA receptors. J Neurosci.

[37] Bernard-Trifilo JA (2005). Integrin signaling cascades are operational in adult hippocampal synapses and modulate NMDA receptor physiology. J Neurochem.

[38] Lin B, Arai AC, Lynch G, Gall CM (2003). Integrins regulate NMDA receptor-mediated synaptic currents. J Neurophysiol.

[39] Takagi J, Petre BM, Walz T, Springer TA (2002). Global conformational rearrangements in integrin extracellular domains in outside-in and inside-out signaling. Cell.

[40] Mould AP, Humphries MJ (2004). Regulation of integrin function through conformational complexity: not simply a knee-jerk reaction?. Current opinion in cell biology.

[41] Tan CL (2011). Integrin activation promotes axon growth on inhibitory chondroitin sulfate proteoglycans by enhancing integrin signaling. J Neurosci.

[42] Wu Y, Chen L, Zheng PS, Yang B (2002). B. beta 1-Integrin-mediated glioma cell adhesion and free radical-induced apoptosis are regulated by binding to a C-terminal domain of PG-M/versican. J Biol Chem.

[43] Schaller MD (1994). Autophosphorylation of the focal adhesion kinase, pp125FAK, directs SH2-dependent binding of pp60src. Molecular and cellular biology.

[44] Xing Z (1994). Direct interaction of v-Src with the focal adhesion kinase mediated by the Src SH2 domain. Molecular biology of the cell.

[45] Groc L (2007). NMDA receptor surface trafficking and synaptic subunit composition are developmentally regulated by the extracellular matrix protein Reelin. J Neurosci.

[46] Gogolla N, Caroni P, Luthi A, Herry C (2009). Perineuronal nets protect fear memories from erasure. Science.

[47] Philpot BD, Sekhar AK, Shouval HZ, Bear MF (2001). Visual experience and deprivation bidirectionally modify the composition and function of NMDA receptors in visual cortex. Neuron.

[48] Lander C, Kind P, Maleski M, Hockfield S (1997). A family of activity-dependent neuronal cell-surface chondroitin sulfate proteoglycans in cat visual cortex. J Neurosci.

