# Supplementary material for: Hyaluronic acid based extracellular matrix regulates surface expression of GluN2B containing NMDA receptors
Source: Sci Rep. 2017 Sep 8;7:10991. doi: 10.1038/s41598-017-07003-3 (PMC5591221; doi:10.1038/s41598-017-07003-3)
Supplement: Supplementary file 1 — Supplementary information [file 41598_2017_7003_MOESM1_ESM.pdf]

Supplementary Material

Hyaluronic acid based extracellular matrix regulates surface expression of GluN2B containing NMDA receptors

Barbara Schweitzer[1], Jeet Singh[1], Anna Fejtova[2,3], Laurent Groc[4], Martin Heine[5]*& Renato Frischknecht[1,6,7]*

[1] Leibniz Institute for Neurobiology, Dept. for Neurochemistry and Molecular Biology, Brenneckestr. 6, Magdeburg 39118 Germany

[2] RG Presynaptic Plasticity, Leibniz Institute for Neurobiology, Magdeburg, Germany

[3] Molecular Psychiatry, Department of Psychiatry and Psychotherapy, University Hospital, Friedrich-Alexander-University Erlangen-Nuremberg, Erlangen, Germany

[4] Centre National de la Recherche Scientifique, Interdisciplinary Institute for Neuroscience, UMR 5297, 33000 Bordeaux, France.

[5] RG Molecular Physiology, Leibniz Institute for Neurobiology; Brenneckestr. 6, Magdeburg 39118 Germany

[6] Center for Behavioral Brain Sciences, D-39118 Magdeburg, Germany

[7] Department of Biology, Animal Physiology, University of Erlangen-Nuremberg, Staudtstrasse 5, 91058 Erlangen, Germany

* Correspondence to: renato.frischknecht@fau.de or martin.heine@lin-magdeburg.de

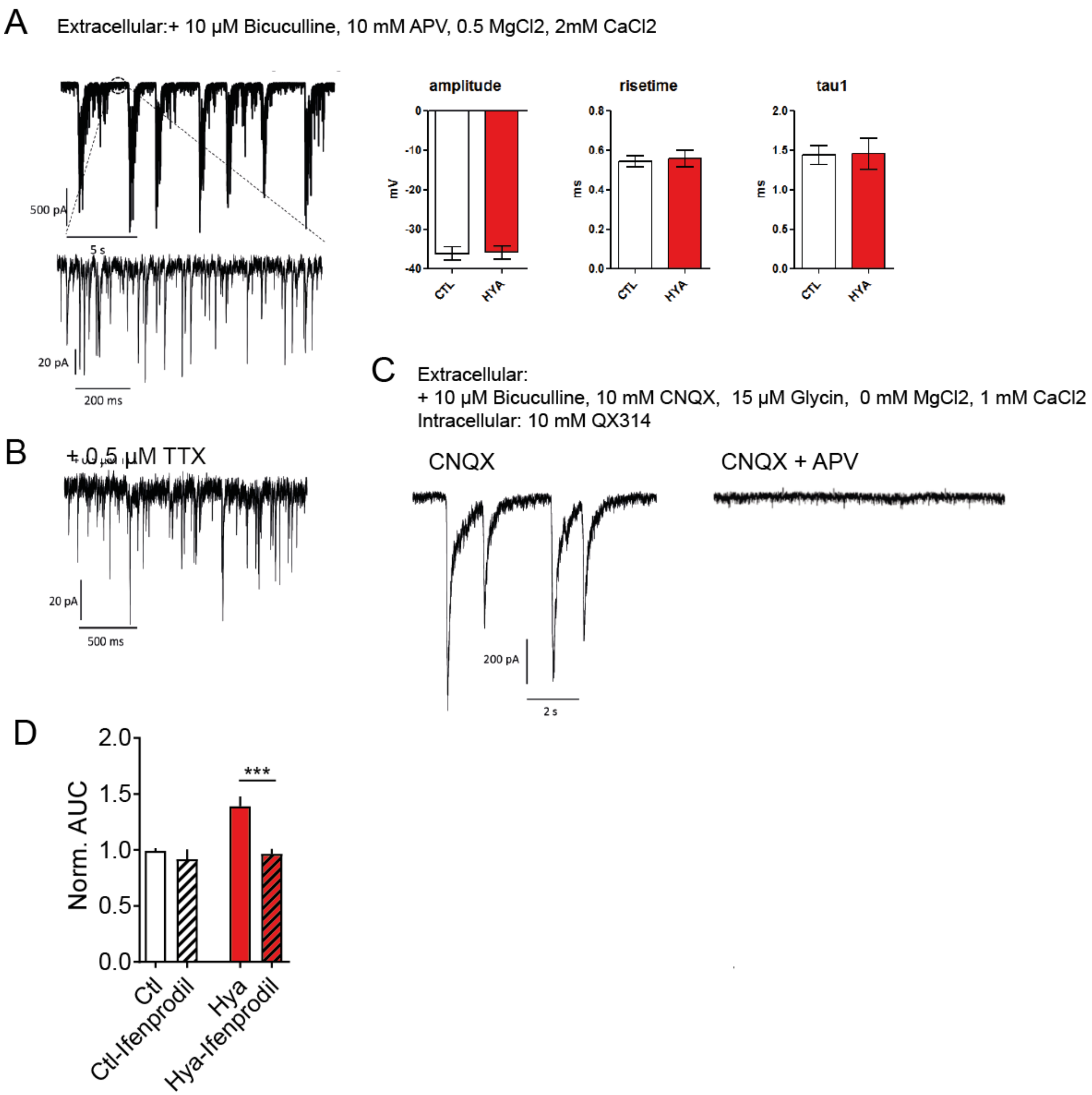

Supplementary Figure S1: Isolation of NMDA receptor mediated currents A) sEPSCs under control conditions, driven by AMPARs and NMDA receptors. Large burst-like events alternated with periods with single events (see magnification) which were analyzed. There was no change in amplitude, rise time or decay time (tau1) in these AMPAR dominated events. **B)** mEPSCs under control conditions. **C)** sEPSCs in presence of CNQX, driven by NMDA receptors. Single peaks with large amplitudes were detected and analyzed, which were abolished by APV. **D)** Ifenprodil had no significant effect on charge transfer before hyaluronidase treatment (Ctl: 1,0 ± 0.02; Ctl-Ifenprodil: 0.92 ± 0,09; Hya: 1.39 ± 0,09; Hya-Ifenprodil: 0.96 ± 0.05, P = 0.0006, Unpaired Student’s t-test).

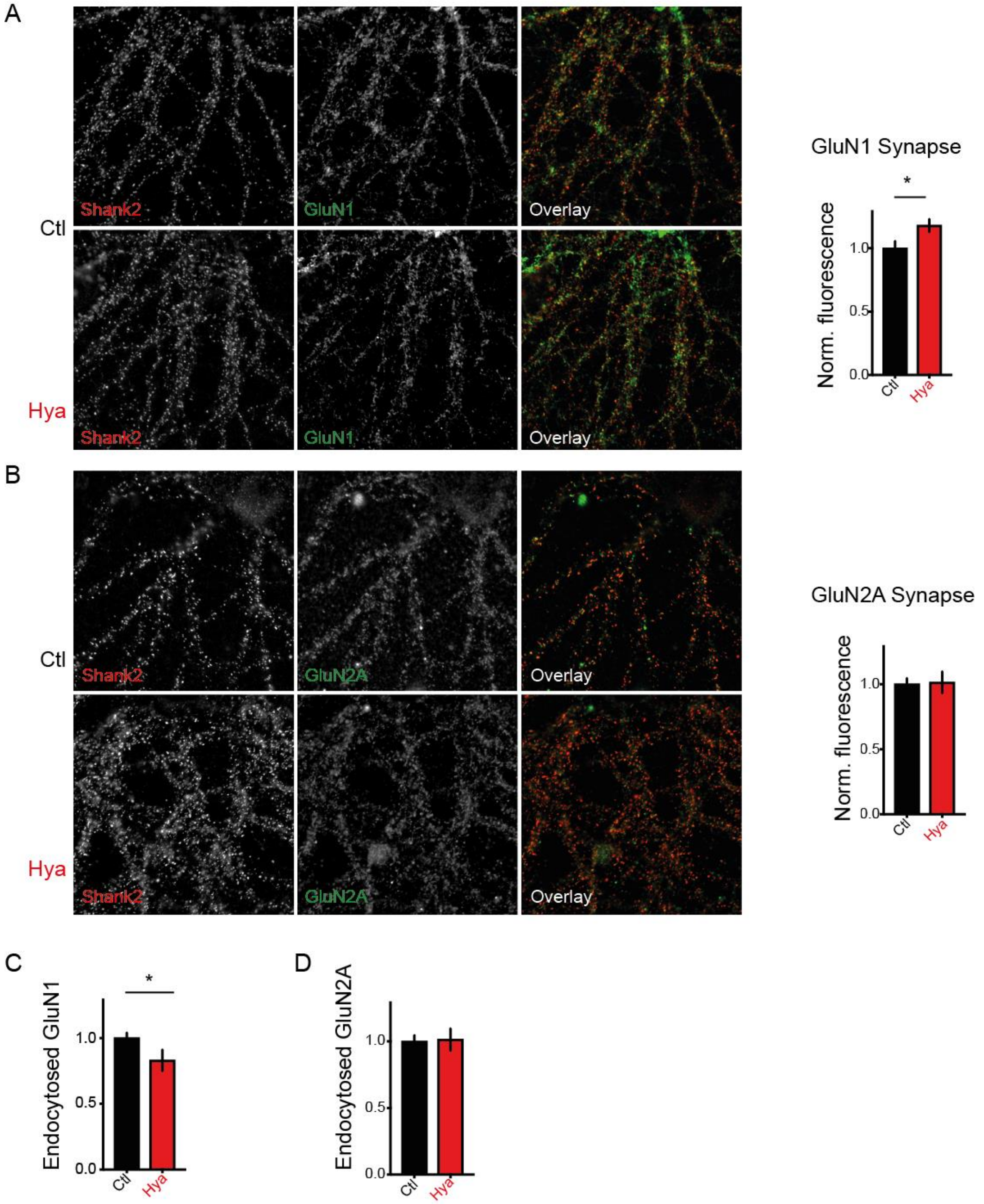

Supplementary Figure S2: Regulation of surface expression of GluN1 and GluN2A subunits. A) Dissociated hippocampal neurons were co-stained for surface expressed GluN1 subunit of the NMDA receptor and the excitatory synapse protein shank2. Quantification of fluorescence intensity at shank2 positive synapses revealed a significant increase of GluN1 subunit after 12 h hyaluronidase treatment (Ctl: 1.00 ± 0.06, n = 28; Hya: 1.18 ± 0.09, n = 27, P = 0.024, Unpaired Student's t-test). **B)** Quantification of synaptic GluN2A subunit co-stained with shank2 revealed no difference between Hya treated and control cells (Ctl: 1.00 ± 0.05, n = 10; Hya: 1.01 ± 0.08, n = 10). C+D) Quantification of endocytosed GluN1 and GluN2A subunits revealed decreased endocytosis for GluN1 but no change for GluN2A after hyaluronidase treatment (GluN1 Ctl: 1.00 ± 0.04, n = 13; Hya: 0.82 ± 0.08 n = 6, P = 0.04; GluN2A Ctl: 1.00 ± 0.08, n = 10; Hya: 1.09 ± 0.10 n = 8; Unpaired Student's t-test)**.**
